# Deep roots through time and crops: insight from five seasons at DeepRootLab

**DOI:** 10.1111/nph.71065

**Published:** 2026-03-13

**Authors:** Eusun Han, Corentin Clément, Weronika Czaban, Abraham George Smith, Dorte Bodin Dresbøll, Kristian Thorup‐Kristensen

**Affiliations:** ^1^ Department of Plant and Environmental Sciences University of Copenhagen Højbakkegård Allé 13 DK‐2630 Taastrup Denmark; ^2^ Department of Agroecology Aarhus University Blichers Allé 20 DK‐8830 Tjele Denmark; ^3^ Agriculture and Food CSIRO PO Box 1700 Canberra 2601 ACT Australia; ^4^ Department of Computer Science University of Copenhagen Universitetsparken 1 DK‐2100 Copenhagen Denmark

**Keywords:** deep learning, deep roots, high throughput, perennial crops, root facility, root phenotyping

## Abstract

- Deep‐rooted crops accessing water and nutrients from deep soil layers enhance the resource base for crop production. However, studying these roots in field conditions is labour‐intensive, limiting research scope.
- We established a field root research facility with 48 plots for replicated experiments. The facility includes 144 6‐metre‐long minirhizotron tubes and an AI‐based pipeline for rapid root trait analysis. We also attempted to install access tubes and customized ingrowth core production for less‐invasive root activity determination.
- Our study revealed significant differences in deep root density among species, particularly at depths of 2.5 to 4.5 m, over 5 years. The less‐invasive studies using ingrowth cores reached depths of 4.2 m. Nutrient tracer ^15^N analysis showed marked differences in deep root activity among crop species. Time domain reflectometry sensors indicated varying water depletion in deeper soil layers, influenced by crop species and root growth patterns.
- We established a field facility for studying deep root growth and function, demonstrating its effectiveness in analysing diverse deep‐rooted plant species. This facility provides an ideal platform for conducting meaningful research in deep soil layers, yielding statistically and biologically significant results for agricultural applications.

## Introduction

No universal consensus has been reached on the pertinence of deep root development of crop plants for increased resource acquisition for food production (Thorup‐Kristensen *et al*., 2020a). This has stemmed from the lack of detailed knowledge on the process of development and functioning of deep roots due to the difficulty in extending the research deeper into the arable subsoil under field conditions (Gregory *et al*., 2022). The requirements for labour, cost and time to observe or sample the living roots in field conditions are high; therefore, often it acts as a bottleneck for deep root studies. Alternatively, semi‐field (Van De Geijn *et al*., 1994; Svane *et al*., 2019b) and rhizobox‐based facilities (Thorup‐Kristensen *et al*., 2020b) have demonstrated the potential to study root systems for resource acquisition and root–soil interactions (Han *et al*., 2024; e.g. temporal root growth cessation). Such facilities have the advantage of easier access and preinstalled equipment, such as water sensors, rhizotron tubes or porous cups for soil water extraction. Furthermore, they may allow a higher degree of control of soil and climatic factors, for example drought conditions. While such approaches can offer more stable root measurements for detailed mechanisms at the root–soil interface, they do in many ways not fully represent the real field conditions.

In field conditions, root researchers must work with the natural soil with a high degree of physicochemical heterogeneity (Kautz *et al*., 2013). Deep installation of devices for measurements requires mechanical drilling, which is costly and logistically demanding, sometimes limiting the achievable sampling depth. The time‐and labour‐intensive nature of *in situ* root studies often constrains the experimental design – particularly the number of treatments, sampling times and replicates – depending on available resources and personnel. For instance, using the profile wall method, recording the rooting density up to 2 m of soil depth for four to five times during the season was possible only with two field plots per treatment (Huang *et al*., 2020; Han *et al*., 2021a). Similarly, soil sampling that requires arduous and time‐consuming root extraction can also limit the number of temporal and spatial observation points. For example, the root length density (RLD) measurement carried out from the soil cores was derived from two replicates only when root washing was involved (Kirkegaard *et al*., 2015). Four replicates were investigated when indirect measurement via root counting was carried out (Wasson *et al*., 2014). Such limitations caused by time‐consuming sample collection can be solved with permanent structures for root measurements that lead to nondestructive sampling processes.

It is challenging to acquire high‐time resolution data on root growth. The destructive samplings may provide detailed answers at specific time points, but they still rely on heavy use of labour for data collection. An alternative is the minirhizotron method (Rewald & Ephrath, 2013). It still requires the laborious installation of the minirhizotron tubes within each plot, but when this is done, it allows a rapid capture of root images. This leads to easier root observation in field conditions, for example comparison of different crop species (Kristensen & Thorup‐Kristensen, 2004) or cropping systems (Båth *et al*., 2008, p. 200) at multiple time points. One of the bottlenecks of the method has been extracting the data from the acquired images, which requires labour‐intensive grid‐counting or manual annotation. It has been suggested that it takes *c*. 20 min per meter of grid line (Böhm *et al*., 1977), which poses challenges for studies aiming at quantification of root growth at multiple observation points in time, space and research treatments. Therefore, a more rapid root quantification method with high accuracy on field root images is called for. Deep learning segmentation of root images has been proposed (Han *et al*., 2021b; Smith *et al*., 2022), which is yet to be validated in field conditions with a diverse range of species.

Crops have shown a wide range of deep‐rooting capacities. The major annual crops such as wheat and canola have been shown to establish root depths often to more than 2 m, depending on genotypes, phenology and management practices (Thorup‐Kristensen *et al*., 2009; Han *et al*., 2015; Rasmussen *et al*., 2015; Dresbøll *et al*., 2016). Perennial field crops such as lucerne, chicory and tall fescue are known to grow up to 2–3 m (Weaver, 1926; Han *et al*., 2021a). The emerging perennial grain crops such as intermediate wheatgrass and rosinweed (also known as silphium) are known to be deep‐rooted below 2–3 m of depth (Van Tassel *et al*., 2017; Pugliese *et al*., 2019). However, continuous observation of deep‐rooting perennial crops over a long period has not been reported sufficiently to understand species differences and their season‐to‐season as well as within‐season dynamics. This information can be helpful when genotypic (G: genotype) potential for deep rooting and subsoil exploitation is in question, which is being attempted in semi‐field conditions (Van De Geijn *et al*., 1994; Svane *et al*., 2019b). Choice of crops and time for sowing/harvest (M: management) greatly influence belowground production, and thus resource acquisition potential (Kristensen & Thorup‐Kristensen, 2004; Rasmussen & Thorup‐Kristensen, 2016). A high‐resolution dataset in this regard has not been produced under field conditions. Therefore, such a research platform and facility are urgently called for GxM research (Hunt *et al*., 2021).

Therefore, we built a field facility, called DeepRootLab, that allows deep root studies to more than 4 m depth. Our aim in developing this facility was to generate statistically significant and biologically meaningful datasets with high efficiency. The previously published articles based on the data from DeepRootLab are shown in Table 1.

**Table 1:** Published studies based on the data acquired from DeepRootLab (up to 2023).

Source	Device used	Root depth	Tracers	Crops investigated	Treatments
Han *et al*. (2020)	Ingrowth cores Access tubes	4.2 m	Li, Cs, Se, Sr, Rb	Lucerne	Soil depths Location of labelling Season
Han *et al*. (2022b)	Ingrowth cores Access tubes	2.5 m	^33^P	Intermediate wheatgrass Perennial lupine Rosinweed Curly dock	Crop species Soil depths
Han *et al*. (2022a)	Ingrowth cores Access tubes	4.2 m	^15^N, Cs	Lucerne‐Winter rye Intermediate wheatgrass Perennial lupine Rosinweed‐Winter wheat	Intercropped vs mono‐cropped
Clément *et al*. (2022)	TDR sensors Minirhizotron tubes	2.8 m	^2^H ^18^O	Lucerne Intermediate wheatgrass	Crop species
Czaban *et al*. (2023)	Ingrowth cores Access tubes Minirhizotron tubes	3 m	^15^N, Cs, Se	Sugar beet Chicory	Intercropped vs mono‐cropped

## Materials and Methods

### Facility layout and crops

**Fig. 1:**
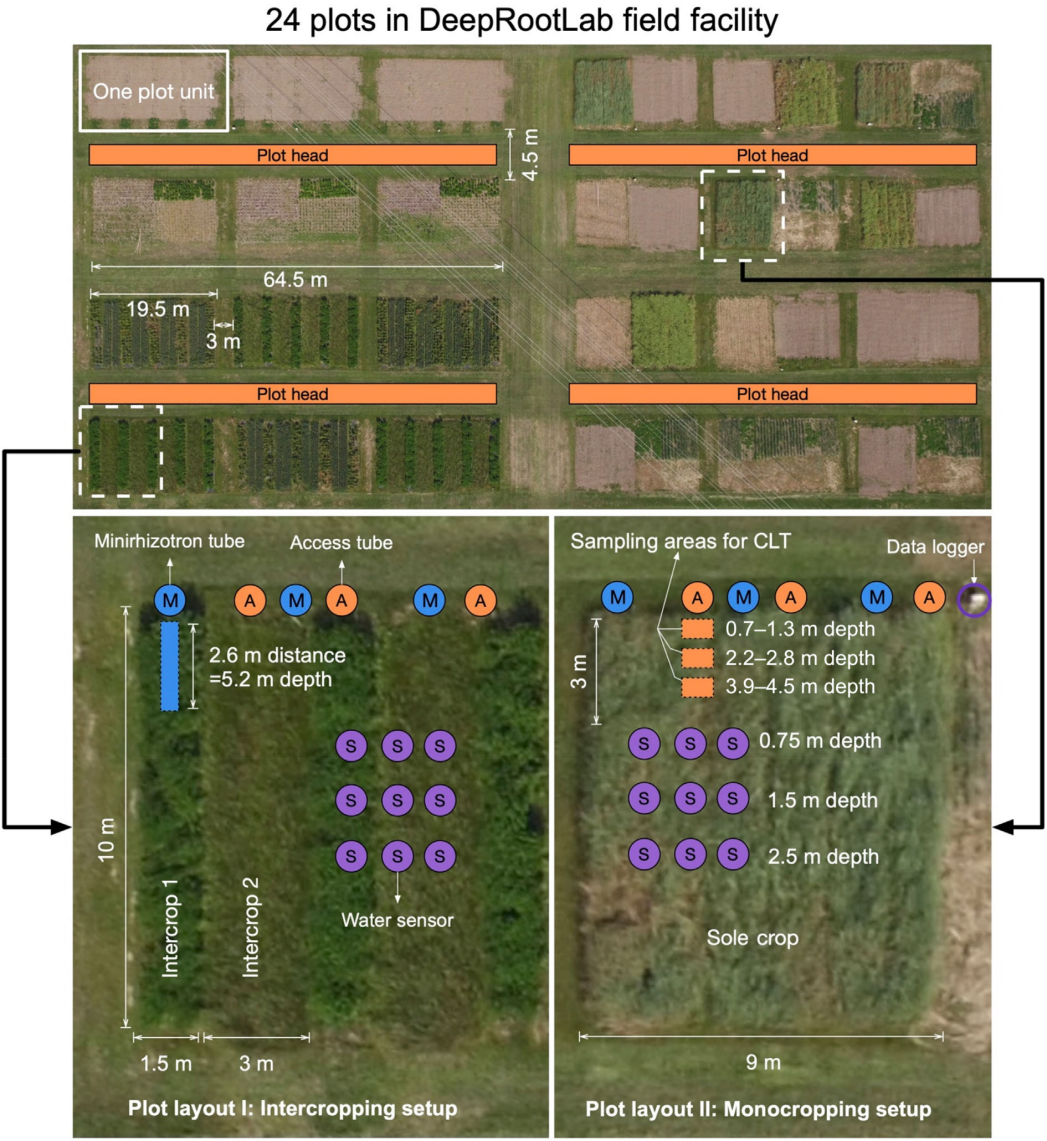
Plot layout at DeepRootLab.

The DeepRootLab facility was established at the experimental station of the University of Copenhagen in Taastrup, Denmark (55°40′ N; 12°18′ E). The soil was classified as Agrudalf. A detailed description of the soil physical and chemical condition at the study site is available in Supporting Information Table S1. Weather conditions recorded at the study site are presented in Fig. S1. In total 24 experimental plots (one plot = 19.5 m × 10 m in size) were laid out in four blocks (Fig. 1). The six plots in each block were laid out in a 3 columns × 2 rows layout, and the plot heads of the two rows were facing each other at a distance of 4.5 m. The distance between neighbouring plots was 3 m. The distance between blocks was 5 m in both column and row‐wise. We divided each plot into two kinds of plot layouts, namely intercropping setup (Layout I) and monocropping setup (Layout II). In Layout I, nine subplots were formed with alternating strips: five 1.5 m‐wide strips for perennial crops (1.5 m in width; 10 m in length) and four strips for annual crops next to each other (3 m in width; 10 m in length) for the study of crops in strip intercropping. In Layout II, the main plot was split into two subplots (9.75 m × 10 m), which can be used for individual crops or treatments. We chose and grew a wide range of perennial and annual crop species that are known to be deep‐rooted as shown in Table 2. No data from intercropping treatments are presented here.

**Table 2:** Crop species at DeepRootLab.

Common name	Scientific name	Sowing date	Sowing density
Lucerne	*Medicago sativa* L.	9 September 2015	2.5 g m^−2^
Intermediate wheatgrass	*Thinopyrum intermedium*	7 April 2016	2.5 g m^−2^
Perennial lupine	*Lupinus perennis* L.	11 April 2016	0.5 g m^−2^
Chicory	*Cichorium intybus* L.	11 April 2016	0.8 g m^−2^
Dyers woad	*Istatis tinctoria*	12 April 2017	0.4 g m^−2^
Mugworth	*Artemisia vulgaris*	28 May 2016	9 plants m^−2^
Rosinweed	*Rosinweed perfoliatum* L.	30 May 2016	9 plants m^−2^
Comfrey	*Symphytum officinale* L.	15 September 2016	9 plants m^−2^
Curly dock	*Rumex crispus* L.	3 May 2017	0.4 g m^−2^
Tall fescue^a^	*Festuca arundinaceae*	30 April 2018, 6 June 2018 and 4 September 2018	Un‐determined
Winter rye	*Secale cereale* L.	24 August 2016	1.15 g m^−2^
Winter wheat	*Triticum aestivum* L.	28 September 2018	2.34 g m^−2^

^a^ Due to a dry condition in spring and summer in 2018, multiple sowing was necessary.

### Installation of equipment

**Fig. 2:**
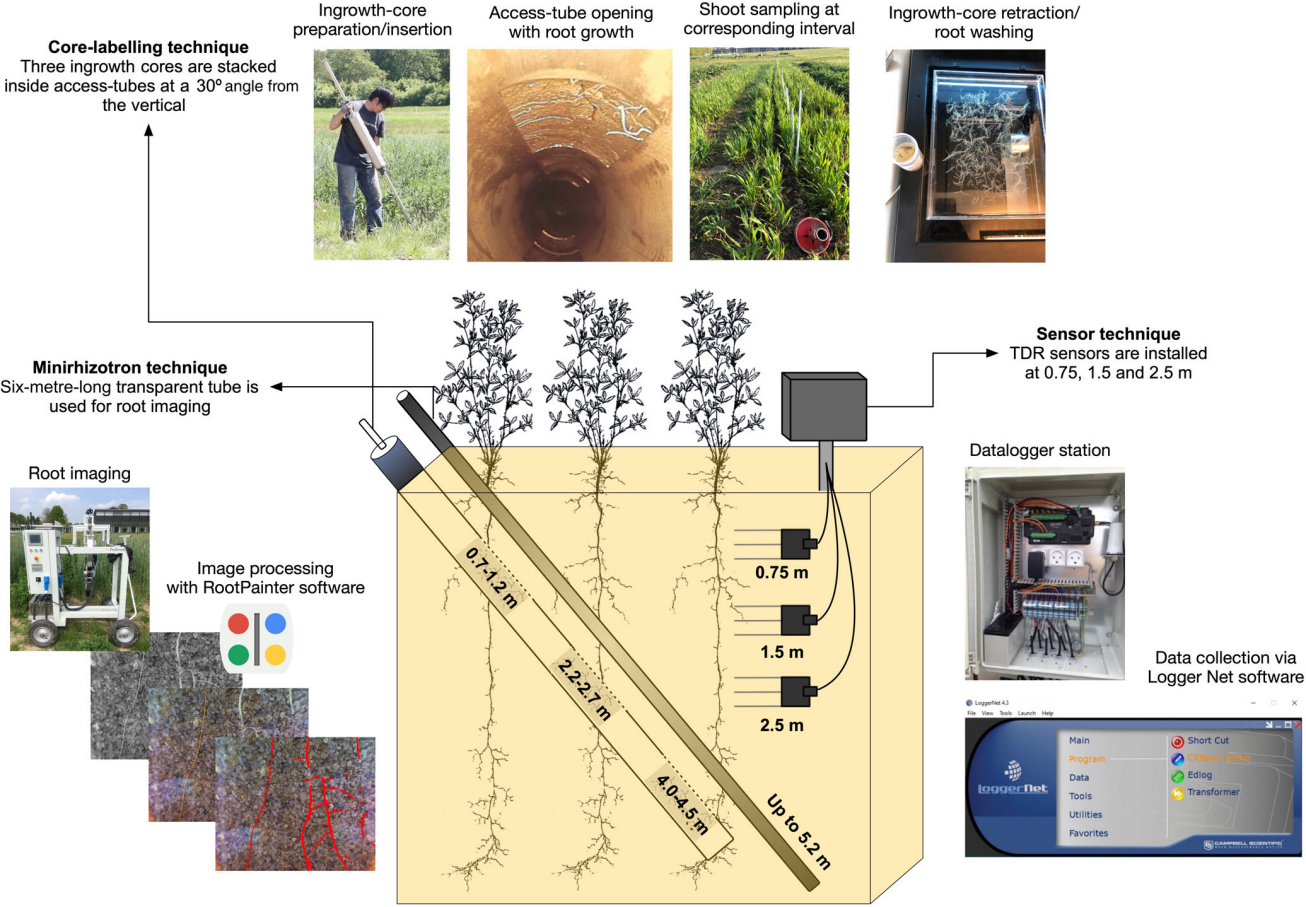
Concept of three techniques and installation of equipment.

We used two types of tubes for deep root study (Fig. 2), both inserted at an angle of 30° from vertical. The minirhizotron tubes are transparent acrylic tubes, 6 m long and have an outer diameter of 70 mm. These tubes were used for nondestructive root measurement with repeated imaging campaigns. The other type is access tubes which are stainless steel tubes (5.85 m in length, 110 mm in outer diameter). Three cut‐out openings were made along the access tubes, which matched soil depths of 0.7–1.2 m, 2.3–2.8 m and 4.0–4.4 m after installation. Ingrowth cores made of stainless steel with a volume of 3931 cm^3^ were made to be filled with soil and inserted into the access tubes, to allow the study of root growth and uptake processes at the different depths. In total 144 minirhizotron and 144 access tubes were inserted at a 30° angle vertically at the heads of each plot (Figs 1, 2). Inserting the tubes took place from late 2015 to early 2016 via auger drilling.

**Time domain reflectometry (TDR) sensors** (Acclima 310S; Acclima Inc., Meridian, ID, USA) were installed for monitoring soil volumetric water content (VWC). In selected plots, three replicated water sensors were installed between late 2017 and early 2018 at each of the depths 0.75, 1.5 and 2.5 m, with measurements being recorded on an hourly basis using four CR6 dataloggers (Campbell Scientific Inc., USA). All TDR sensors were located at least 3 m away from the plot edges (Fig. 1). The cables were buried at 0.3 m to allow the use of agricultural machinery.

We developed an AI‐based software for automated root image analysis called RootPainter (Smith *et al*., 2022). The software features a user‐friendly interface that can be easily operated by researchers without experience in Machine Learning. RootPainter utilizes a convolutional neural network that is built on the U‐Net architecture. After its release, the software was validated for root quantification during destructive sampling (Han *et al*., 2021b).

### Measurement of root growth dynamics

We monitored root growth dynamics of lucerne (2016–2019), intermediate wheatgrass (2016–2019), perennial lupine (2016–2019), mugwort (2016–2019), rosinweed (2017–2019), comfrey (2017–2019), curly dock (2017–2019) and tall fescue (2019–2020) by minirhizotron technique (Tables S2). Multispectral images were taken along the minirhizotron tubes at each 0.05 m‐length interval by a Videometer lab instrument (Videometer A/S, Hørsholm, Denmark). Multispectral images were obtained by using Light‐Emitting Diode (LED) light in five different wavelengths ranging from Ultraviolet A (UVA) to violet, amber, red and Near‐Infrared (NIR) was adjusted to 365, 405, 590, 660 and 970 nm, respectively. The camera system was mounted on a pull‐type wagon equipped with an automated deployment mechanism, which allowed the camera to be lowered to the desired soil depth to capture images at predefined depth intervals during retraction. The meta‐data, namely, plot number (PipeID), camera position (Position), Year, Month, Date and Time (hh‐mm‐sec) of the imaging were formulated into the file names for further identification and data processing (e.g. ‘PipeID_61_Position_442_TS_2019‐08‐23 09.15.40’). In the results shown, the images were converted to pseudo‐RGB images before further analysis. The size of each of the images was 40 mm × 50 mm (width × height) consisting of 2048 × 2448 pixels. From the images, root density was determined as planar root length density (pRLD) – root length per image area (cm cm^−2^; Wacker *et al*., 2025) after training the model using the deep learning software (see the Results section for detailed description). The maximum root depth of each tube was manually determined. For each crop species, we selected one image dataset per year during the crop season. The root depth of each species over accumulative thermal time (°C days) was computed.

### Root activity measurement by core‐labelling technique

From 2017 to 2019, we conducted three experiments (Exp 1, 2 and 3) to compare three crop species for deep root growth and nutrient uptake potential (Table 3). Each experiment included at least two perennial species with relatively earlier sowing dates (lucerne, mugwort and rosinweed) and later sowing dates (chicory, dyers woad and curly dock). In addition, winter cereals or a perennial cereal crop were included for comparisons (winter rye, intermediate wheatgrass and winter wheat).

**Table 3:** Experimental design for three experiments.

		Exp 1	Exp 2	Exp 3
Period		2017 May 4–July 4	2018 May 25–July 25	2019 April 1–May 31
Crop	Early sown perennial	Lucerne (665)^a^	Mugworth (819)	Rosinweed (1100)
	Late sown perennial	Chicory (450)	Dyers woad (368)	Curly dock (762)
	Cereal crops	Winter rye (315)	Intermediate wheatgrass (768)	Winter wheat (249)
Soil depth		0.7–1.2 m 2.2–2.8 m	0.7–1.2 m 2.2–2.8 m	0.0–0.3 m 0.7–1.2 m 2.2–2.8 m 4.0–4.5 m

^a^ The numbers in the brackets indicate the number of days after sowing (DAS).

We designed an experimental workflow called core‐labelling technique (CLT) for measuring deep root uptake (Han *et al*., 2020). Root‐free soil medium labelled with tracers was packed inside the ingrowth cores and inserted into the access tubes. Ingrowth cores were installed at two or three depths, matching the cut‐out openings of the access tubes. We used various tracers; here we present results obtained with ^15^N as a nutrient tracer. For each ingrowth core, 275.24 mg of ^15^NH_4_Cl was prepared in solution and mixed with a subsoil medium (see Table S1 for physical and chemical soil characteristics) before packing the soil into the ingrowth cores. Tracer concentration determined from the shoot biomass was used as an indicator of root activity.

Ingrowth cores were inserted at two depth levels in Exp 1 and 2 (0.7–1.2 m and 2.2–2.8 m), whereas four depth levels were investigated in Exp 3 (0–0.3 m, 0.7–1.2 m, 2.2–2.8 m and 3.9–4.5 m), where an extra ingrowth core was installed directly in the topsoil to complement the three deeper placements using the access tubes. Shoot biomass was sampled twice, at the fourth and eighth weeks after insertion of the ingrowth cores. The dried and powdered samples were analysed for ^15^N enrichment. For each experiment, root growth of each crop species was monitored using minirhizotron technique.

#### Water content measurement by sensors

In this study, soil water content was monitored from June 2018 to June 2020 in three replicated plots for chicory, lucerne and intermediate wheatgrass, and in a single plot for perennial lupin, dyer's woad and oilseed rape as a pilot trial.

### Statistical analysis

The R software (R Development Core Team, 2019) was used for statistical analysis. Linear regression between segmentation and manual counts was performed to compute the R^2^ values as it was affected by increasing annotation time. Root depth dynamics of the eight key species (lucerne, intermediate wheatgrass, perennial lupine, mugwort, rosinweed, comfrey, curly dock and tall fescue) between the years (2016 to 2020) were analysed using Tukey HSD (*P* ≤ 0.05). pRLD of the crop species was compared between the growing seasons regardless of the soil depth. More detailed comparisons within seasons in 2017, 2018 and 2019 between the image campaigns were done for four crop species (lucerne, intermediate wheatgrass, rosinweed and tall fescue). For three experiments conducted, pRLD measured by minirhizotrons was compared between the crop species at 0–0.3 m, 0.3–0.7 m, 0.7–1.3 m, 1.3–2.3 m, 2.3–2.8 m and 2.8–4.0 m of soil depth. RLD and δ^15^N (‰) were compared between the crop species at soil depths of 0–0.3 m, 0.7–1.3 m, 2.2–2.8 m and 3.9–4.5 m within each experiment. Linear regressions of RLD vs δ^15^N (‰) measured from the ingrowth cores, RLD by ingrowth cores vs root length by minirhizotron and root length by minirhizotron vs δ^15^N (‰) were computed combining the data from the nine crop species used in the three experiments. *R* ^2^ values were computed for each crop species.

## Results

### Data generation capability at DeepRootLab

#### Automated root phenotyping

The main data‐gathering operation incurred at the facility has been the root imaging via minirhizotron tubes (Fig. 2). In our setup, we had an efficiency of acquiring one image every 5 s. Consecutive images to 2.3 m to 4.3 m depth per tube were taken. However, depending on the season and crop, the imaging depth was often varied. Root growth for most of the crops was monitored using six minirhizotron tubes (=6 replicates), except for perennial lupine, mugwort and comfrey, which had three replicates, and intermediate wheatgrass with nine replicates. Imaging a tube down to 2.3 m and 4.3 m took 4.5 min and 8.3 min, respectively. Considering the movement and setup of the camera wagon, it was possible to image 36 tubes per day. This allowed multiple imaging campaigns per treatment per season throughout the facility.

The raw image format (Hips: ) from the camera was converted to a more common JPEG format. In our estimate, 5.2 s per image was required for this process. We converted 61 471 images which involved *c*. 60 h. Afterwards, we spent *c*. 10‐h training the dataset using the RootPainter software, which had an efficiency of 0.6 s per image for inference.

Therefore, in our estimate, 11.8 s per image was spent on image capture, processing and model training for segmentation. In total, we processed 61 471 images acquired over 5 yr (2016 to 2020). This required *c*. 200 h for the entire process which is *c*. 25 d – which is equivalent to 5 working days per year.

#### Root activity determination at DeepRootLab

In total, 36, 63 and 96 ingrowth cores were installed in Exp 1, 2 and 3, respectively. In our estimate, three workdays were needed to complete the labelling and packing of 36 ingrowth cores. Insertion/retraction of ingrowth cores into/from the access tubes required ideally three persons and 1–2 d of operation time depending on the weather conditions and occurrence of mechanical disturbance, for example ingrowth cores jammed inside the access tubes.

#### TDR sensor recording

TDR sensors' data were recorded hourly and data withdrawal was done manually using the Logger Net software. Downloaded ‘dat’ files were used to extract VWC data. Soil water content data were averaged daily for this study.

### Automated root segmentation

#### Model performance

**Fig. 3:**
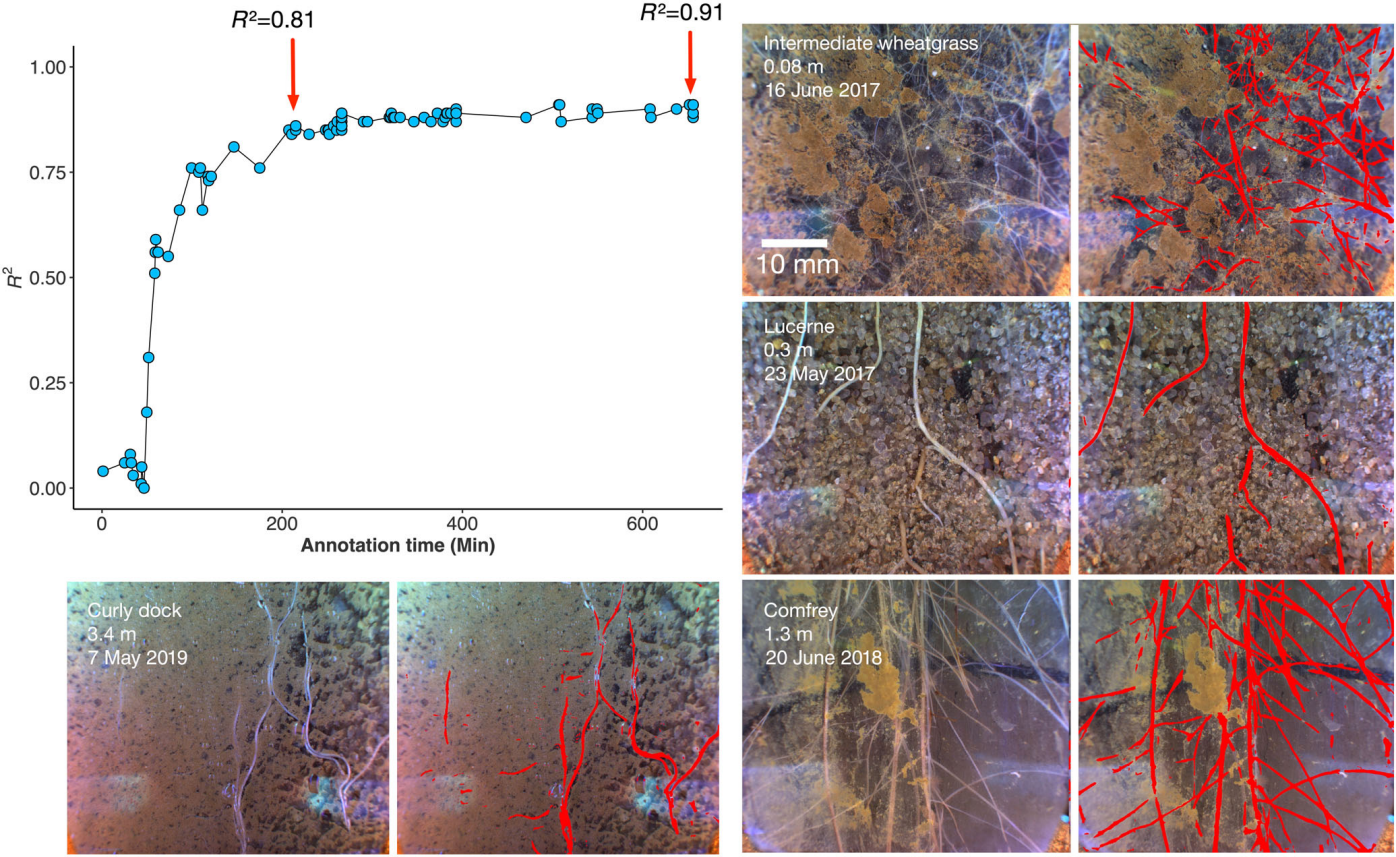
*R* ^2^ values derived from linear regression between segmentation and manual counts as a function of annotation time. Examples of Minirhizotron images showing original images (left column) and the corresponding automated root segmentation results (right column, roots highlighted in red) for different species and depths: intermediate wheatgrass (0.08 m), lucerne (0.3 m), curly dock (3.4 m), and comfrey (1.3 m).

We report the performance of a root segmentation model trained on the root images using the RootPainter software. Manual counts on 200 random images were correlated against the automatic segmentation over the period of annotation/training (Fig. 3). After 200 min of annotation, *R* ^2^ reached 0.81. After *c*. 10 h of corrective annotation, we found an *R* ^2^ of 0.91 with the manual root counted data, showing high root detection accuracy.

#### Training process

**Fig. 4:**
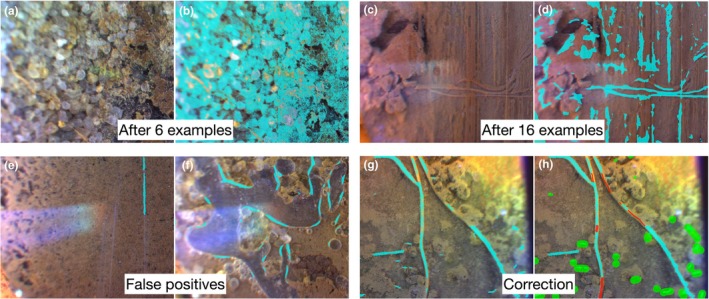
Prediction after six (a, b) and 16 annotations (c, d); false positives – scratches and water bubbles (e, f); corrective annotation to connect the gaps in root segmentation and remove false positives (g, h). Red brush and green brush were used for foreground and background. Prediction is shown in aqua blue.

We modified the established training protocol by Smith *et al*. (2022) – annotation on six examples before initiation of corrective annotation (in which annotators use the prediction by the model as a guidance) as we did not see a clear improvement in prediction (Fig. 4a,b). Therefore, we initiated corrective annotation after 16 clear examples when we observed roots being segmented with a clear shape (Fig. 4c,d). False positives on nonroot objects such as the scratches on minirhizotron tubes (Fig. 4e) and water bubbles (Fig. 4f) prevailed throughout the training process. We focused on making corrections to the false positives. As a result, the model started to exclude the artefacts accurately after the 467th loaded image.

**Fig. 5:**
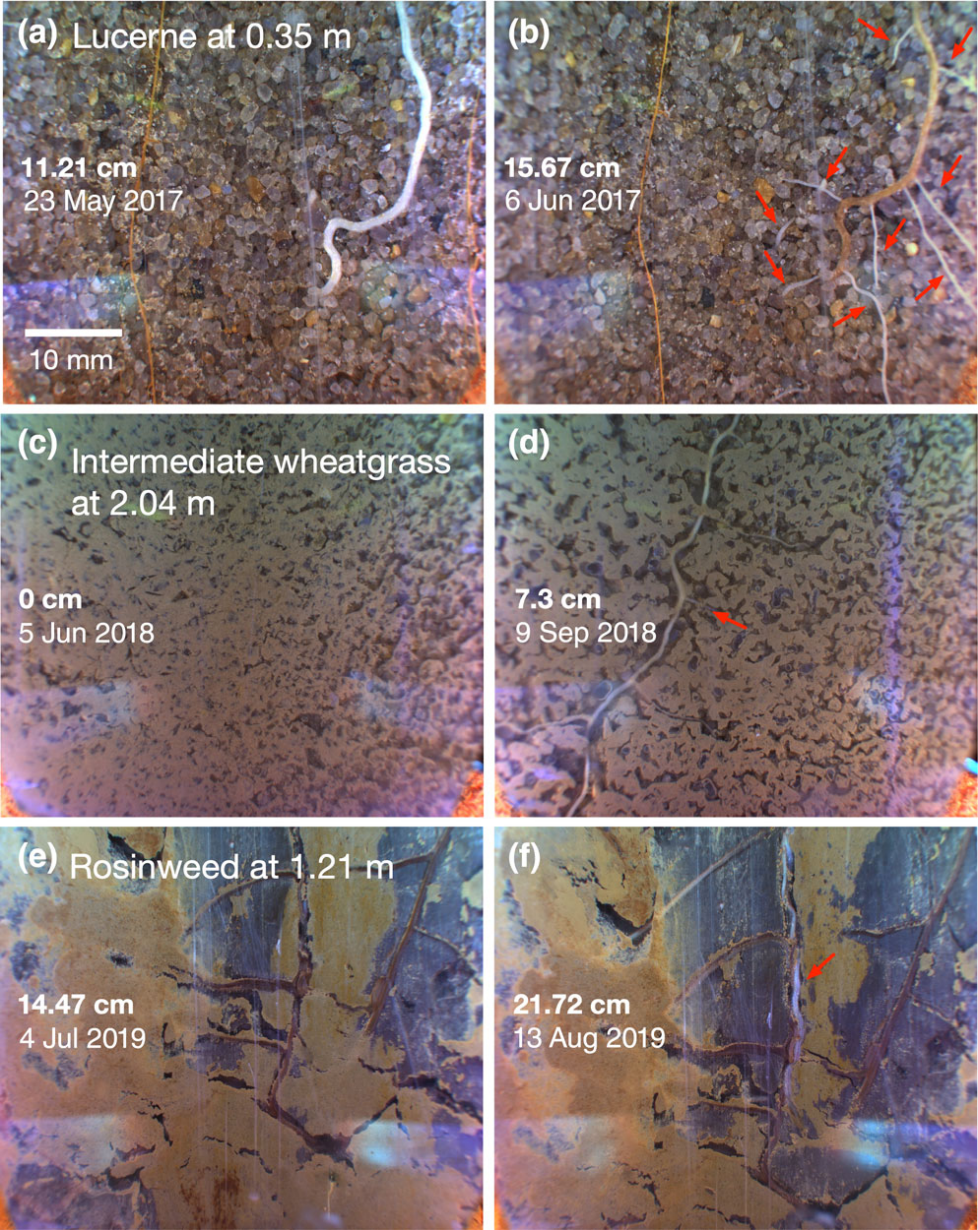
Visually captured seasonal root growth dynamics. Lateral branching of lucerne roots at 0.35 m taken on May 23 (a) and June 6 (b) in 2017. Penetration and lateral branching of intermediate wheatgrass roots into new root depth at 2.04 m taken on June 5 (c) and September 9 (d) in 2018. Penetration of rosinweed roots into pre‐existing root depth at 1.21 m taken on July 6 (e) and August 13 (f) in 2019. Arrows indicate new root growth. Root length (cm) was derived from segmentation, see the changes in root length between Figure a to b, Figure c to d and Figure e to f.

We also occasionally observed false negatives that is roots not predicted as roots after corrective annotation was initiated – for example, one single root was segmented as separate parts (Fig. 4g: at 162^nd^ image). Whenever applicable, we connected the gaps between the separate segmentations as one single root (Fig. 4h). These efforts improved the predictions substantially towards the end of training. Most importantly, the trained model accurately predicted root growth when (1) old branches produced laterals (Fig. 5a,b); (2) roots penetrated into a new depth (Fig. 5c,d); and (3) roots penetrated into a depth with old roots (Fig. 5e,f) that provided quantitative information on the increment of root density (in cm) between two imaging intervals.

### Root depth and penetration rate of 8 perennial crops

**Fig. 6:**
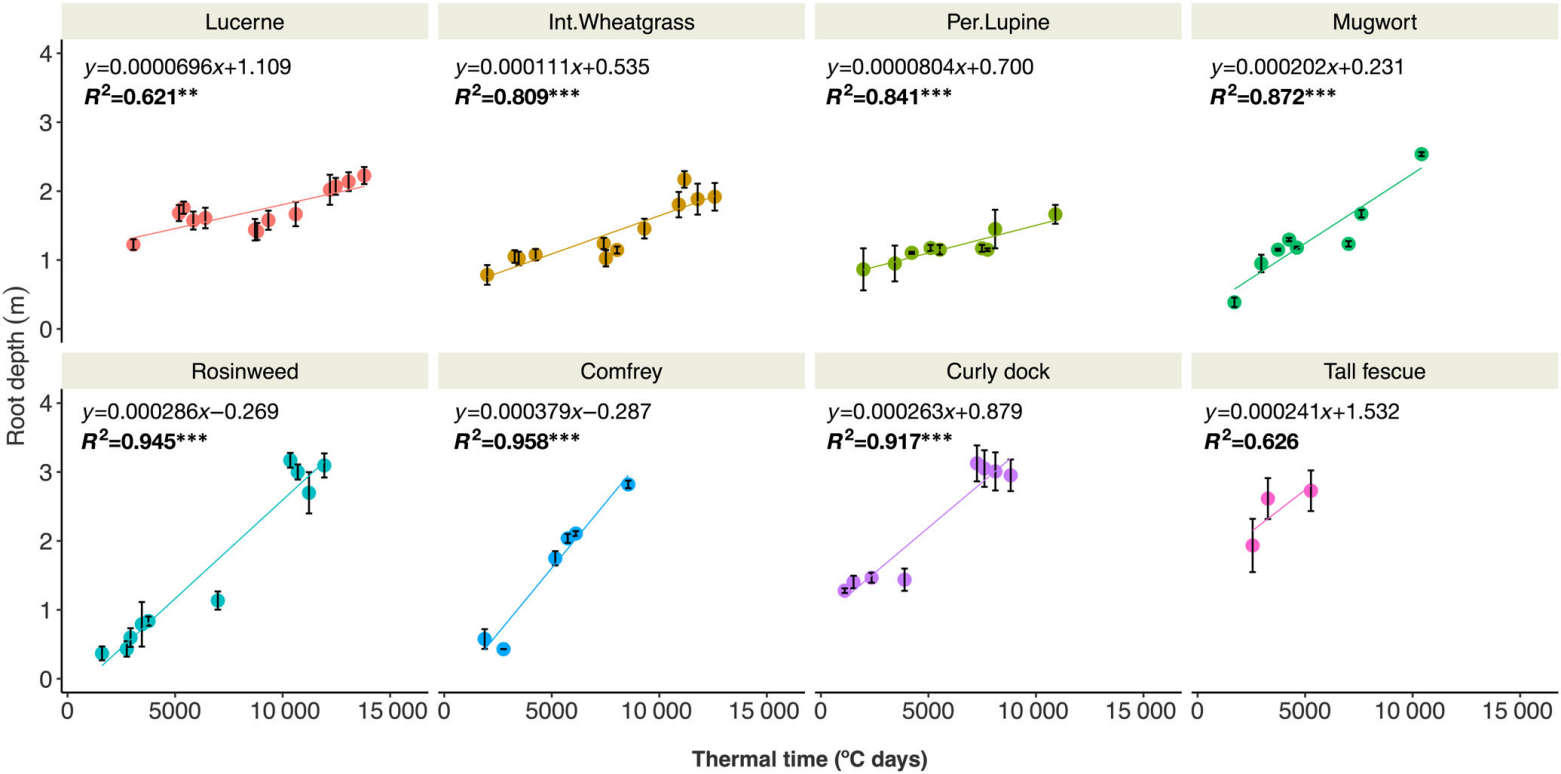
Root depth dynamics of perennial species at DeepRootLab facility measured over thermal time (°C days) from 2016 to 2020. Mean and SE (± one) are shown plotted over linear regression lines.

We determined root depth dynamics of the eight perennial crop species (Fig. 6). Overall, there was a clear trend of increasing average root depth over the years by all measured crop species. The root penetration rate computed by regression analysis between root depth over the thermal time (°C days) was slowest for lucerne (0.07 mm °C d^−1^) followed by perennial lupine (0.08 mm °C d^−1^), intermediate wheatgrass (0.1 mm °C dys^−1^), mugwort (0.2 mm °C d^−1^), tall fescue (0.24 mm °C d^−1^), curly dock (0.26 mm °C d^−1^), rosinweed (0.29 mm °C d^−1^) and comfrey (0.38 mm °C d^−1^). Maximum root depth – the deepest point of root observation was shown by curly dock and rosinweed in 2019 at 3.8 m, followed by tall fescue (3.5 m), intermediate wheatgrass and comfrey (2.9 m), lucerne (2.7 m) and mugwort (2.6 m).

### Yearly dynamics in root density

**Fig. 7:**
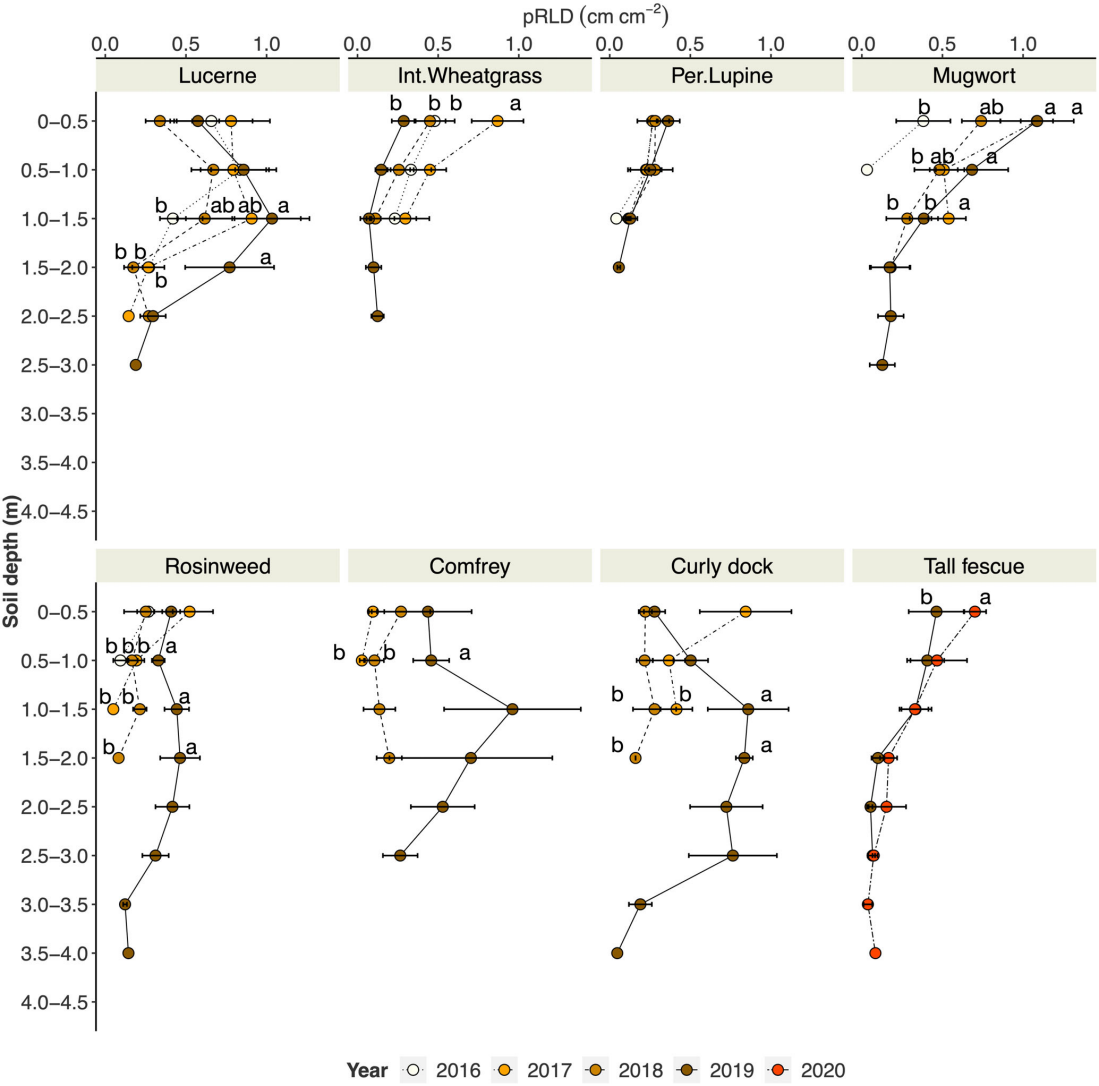
Yearly root growth dynamics of lucerne, intermediate wheatgrass, perennial lupine, mugwort, rosinweed, comfrey, curly dock, tall fescue expressed as planar root length density (pRLD; cm cm^−2^). Small letters indicate significant differences between the year (Tukey HSD; *P* ≤ 0.05).

We compared the root density (measured as pRLD (cm cm^−2^) of eight perennial species between crop seasons at each 0.5‐m interval to 4.0 m of soil depth (Fig. 7)). Overall, root length generally increased as the crops grew older. The effect was most pronounced below 0.5–1.0 m of soil depth, except for mugwort and tall fescue. Intermediate wheatgrass exhibited greater root length in its second growing season (in 2017), especially in the upper soil layer (0–0.5 m), but its root density decreased over the following 2 yr. Perennial lupine did not show any effect of crop age on root length. All crop species revealed deeper rooting from the first to the final point of observation (note the sowing date and stand age by referring to Table 3).

### Seasonal root growth dynamics

**Fig. 8:**
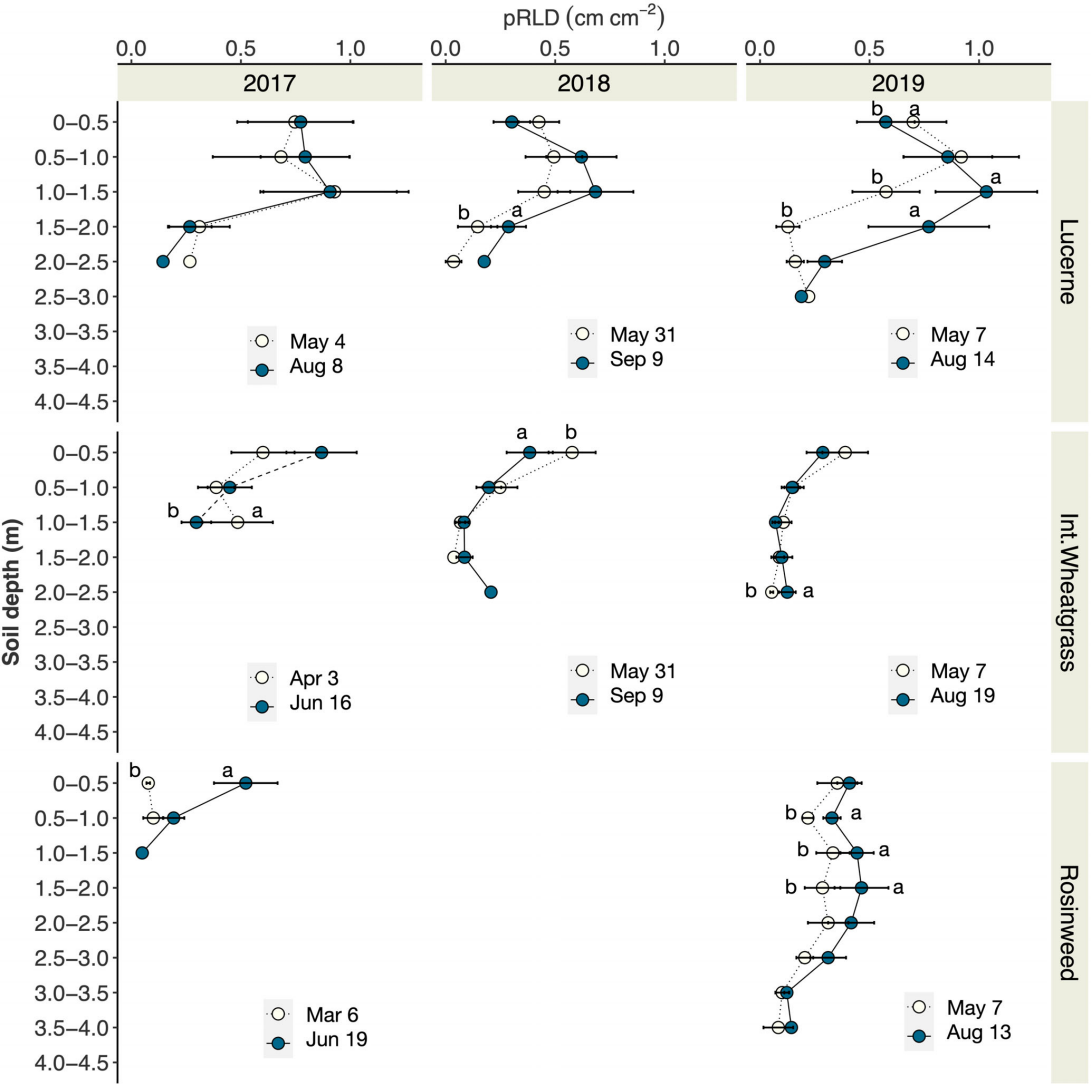
Seasonal root growth of lucerne, intermediate wheatgrass, rosinweed and tall fescue measured in 2017, 2018 and 2019 expressed as planar root length density (pRLD; cm cm^−2^). Small letters indicate significant differences between the image campaign (Tukey HSD; *P* ≤ 0.05).

We compared the root density of lucerne, intermediate wheatgrass and rosinweed at each depth‐level between the two observation times within the crop seasons in 2017, 2018 and 2019 (Fig. 8). Root density of lucerne remained unchanged in 2017 between the observation points, but in the following 2 yr, an increase in root density was shown below 1.0–1.5 m soil depth. In 2019, a significant decrease in root density was observed in the topsoil. Intermediate wheatgrass showed little effect of measurement time, but with a tendency to declining root density later in the season. Rosinweed exhibited an increase in root density within the season in 2017 (0–0.5 m) and 2019 (0.5–1.0, 1.0–1.5, 1.5–2.0 m).

### Crop comparison for deep root development

**Fig. 9:**
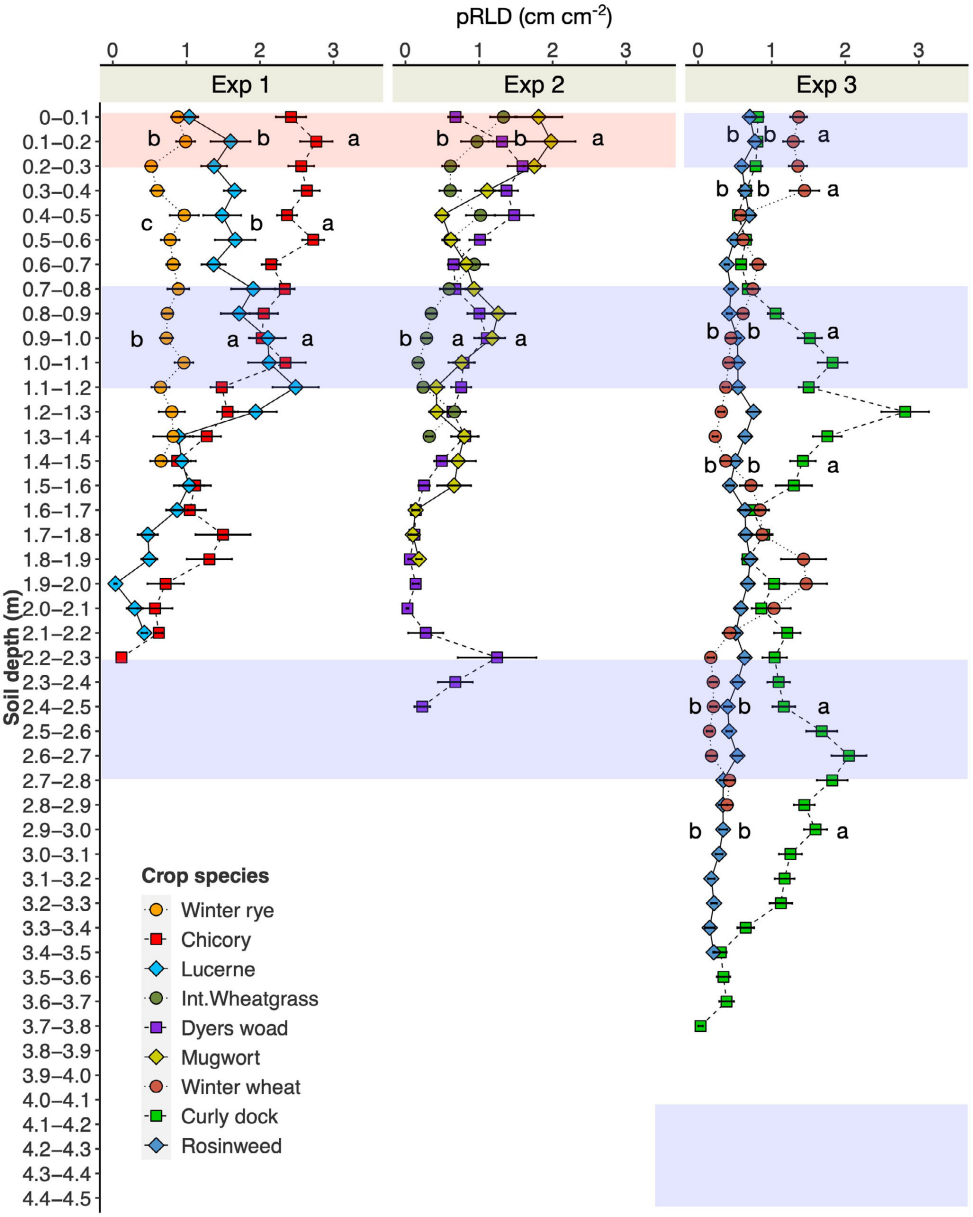
Planar root length density (pRLD) (cm cm^−2^) measured at Exp 1, 2 and 3 by minirhizotron imaging and segmentation. The images were acquired 8 wk after Ingrowth‐core insertion. Purple strips indicate the depth range for ingrowth‐core measurement. No ingrowth core was inserted in the white and red strips. Small letters indicate significant difference between the crop species within the depth range (red, white and purple) at each experiment (Tukey HSD: *P* ≤ 0.05).

Using minirhizotron technique, we observed a clear difference in root depth among the compared crop species (Fig. 9). In Exp 1, chicory showed the deepest roots at 2.2–2.3 m depth followed by lucerne (2.1–2.2 m) and winter rye (1.4–1.5 m). In general, winter rye had the lowest and chicory the highest root density along the soil depth profile. Root density of chicory was greater than lucerne in the upper soil layers (0–0.7 m).

In Exp 2, dyers woad showed the deepest roots (2.4–2.5 m), followed by mugwort (1.8–1.9 m), and intermediate wheatgrass (1.3–1.4 m). In the topsoil, root density of mugwort was greater than that of other crops. There was some variability with depth, but in general, intermediate wheatgrass showed lower root densities than dyers woad and mugwort.

We found roots of curly dock to 3.8 m depth in Exp 3. Winter wheat and rosinweed grew roots to 2.8–2.9 m and 3.3–3.4 m, respectively. Except in the topsoil layer, curly dock exhibited a higher root density compared with rosinweed and winter wheat.

### Crop comparison for deep root activity

**Fig. 10:**
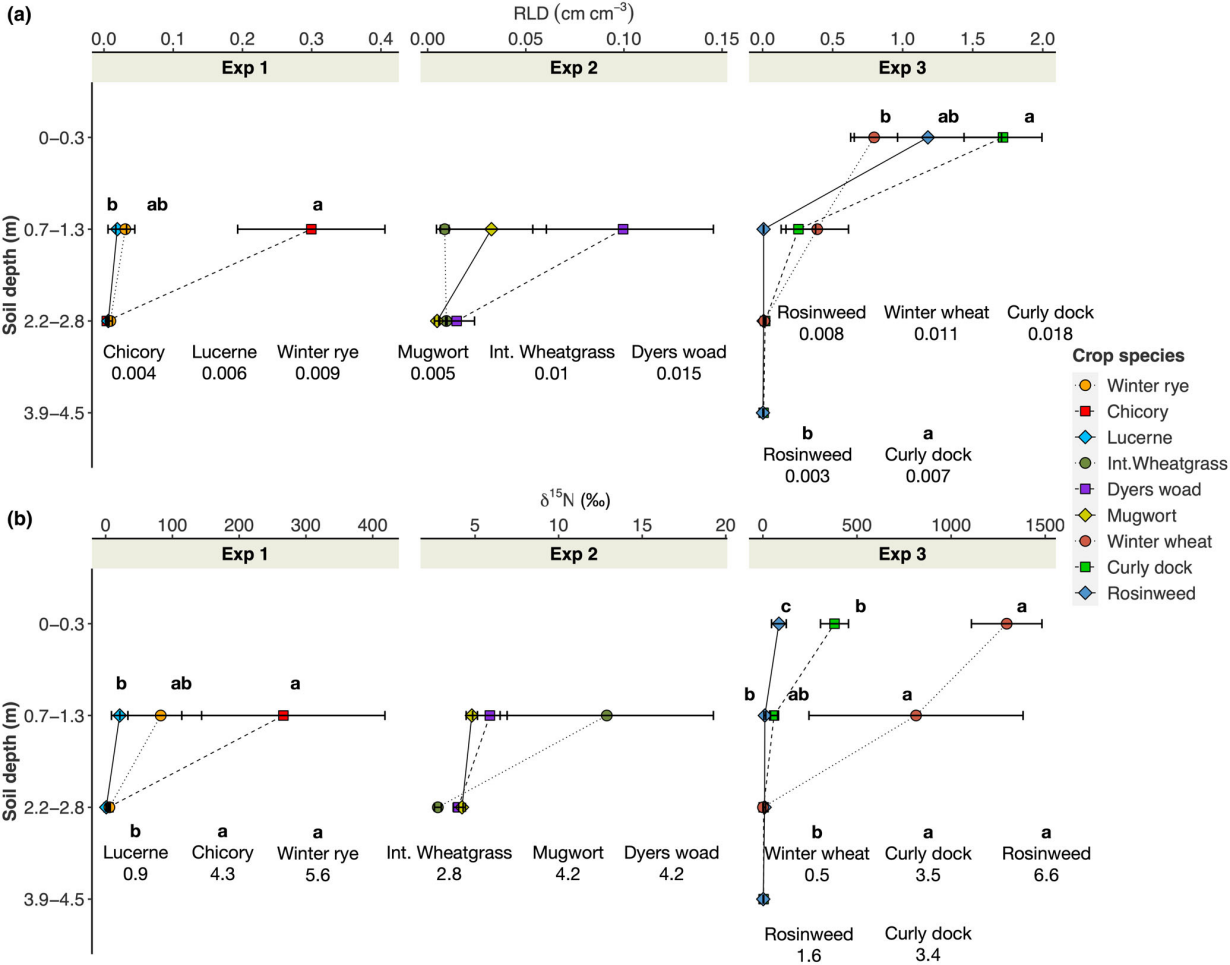
Root length density (a; RLD; cm cm^−3^) and δ^15^N (b; per mill) concentration measured in Exp 1, 2 and 3. Small letters indicate significant differences between the crop species within the soil depth (Tukey HSD: *P* ≤ 0.05). For 2.2–2.8 m and 3.9–4.5 m depths, RLD and δ^15^N values are shown in numbers.

We compared deep root activity of three crop species by inserting ingrowth cores packed with ^15^N‐labelled soil in Exp 1, 2 and 3. Two parameters were measured in all experiments, namely RLD (cm cm^−3^) from the root extraction from the soil in the ingrowth cores (Fig. 10a) and δ^15^N (‰) concentration measured from aboveground biomass (Fig. 10b).

In Exp 1, the ingrowth cores were inserted at 0.7–1.2 m and 2.2–2.8 m depth levels. At 0.7–1.2 m, chicory exhibited greater RLD and δ^15^N compared with lucerne, while intermediate results were shown by winter rye. No difference in RLD was found between the crop species at 2.2–2.8 m. However, δ^15^N of winter rye and chicory was greater than lucerne at this depth.

Ingrowth cores were inserted at the same depth levels in Exp 2. No meaningful difference in RLD and tracer concentration was found in Exp 2. Overall values of RLD and δ^15^N were low, especially at 0.7–1.2 m.

In Exp 3, the following four depth levels were investigated: 0–0.3 m, 0.7–1.2 m, 2.2–2.8 m and 4.0–4.5 m. In the topsoil layer (0–0.3 m), RLD of winter wheat was significantly lower than curly dock, but δ^15^N of winter wheat was substantially greater than both other crops. No difference in RLD was found at 0.7–1.2 m, yet winter wheat exhibited greater δ^15^N compared with rosinweed. At 2.2–2.8 m, greater δ^15^N was found for rosinweed and curly dock compared with winter wheat, whereas no difference in RLD was found. RLD of curly dock was greater than rosinweed at 2.2–2.8 m. A similar trend was found for δ^15^N at 4.0–4.5 m; however, the difference was not significant.

### Validating measurements

**Fig. 11:**
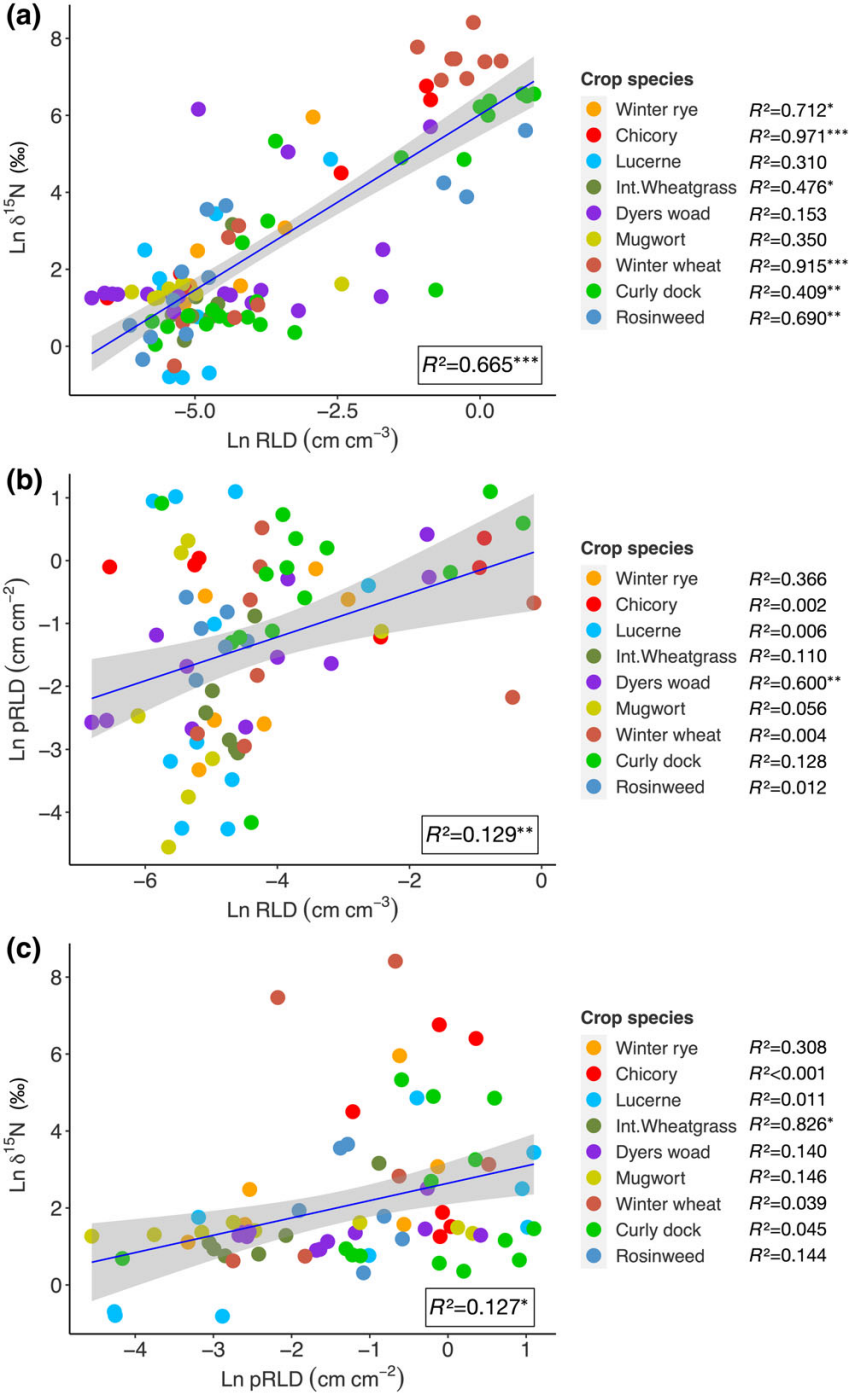
Linear regressions between root length density (RLD) inside ingrowth cores and δ^15^N concentration (a, RLD vs δ^15^N); RLD and pRLD measured by minirhizotron (b, RLD vs pRLD); root length measured by minirhizotron and δ^15^N concentration (c, pRLD vs δ^15^N). *R* ^2^ values as a combined dataset for each experiment were shown inside the plots, whereas *R* ^2^ values for individual species are shown in legends.

Regression analysis between the root density found inside the ingrowth cores and tracer uptake (δ^15^N) measured from aboveground biomass showed a significant relationship (*P* ≤ 0.001) with *R* ^2^ values of 0.67 when all data from the three experiments were combined (Fig. 11a). When the same analysis was performed for each crop species, it revealed strong correlations for chicory (*R* ^2^ = 0.97; *P* ≤ 0.001) and winter wheat (*R* ^2^ = 0.92; *P* ≤ 0.001). Except for lucerne, dyers woad and mugwort, the relationship between RLD and δ^15^N was found to be significant for the rest of the crops.

We performed linear regression analysis between root density found inside the ingrowth cores and root density measured by minirhizotron imaging (Fig. 11b). The relationship was found to be significant (*P* ≤ 0.01), however, with a small *R* ^2^ value (0.13). When performed on each crop species, only dyers woad showed a significant relationship with a moderate *R* ^2^ value (0.6; *P* ≤ 0.01).

Similarly, a weak but significant relationship (*R* ^2^ = 0.13; *P* ≤ 0.05) was found between root density observed by minirhizotron and tracer uptake (δ^15^N; Fig. 11c). Only intermediate wheatgrass exhibited a significant relationship between these two measurements (*R* ^2^ = 0.83; *P* ≤ 0.05*).

### Water measurement

**Fig. 12:**
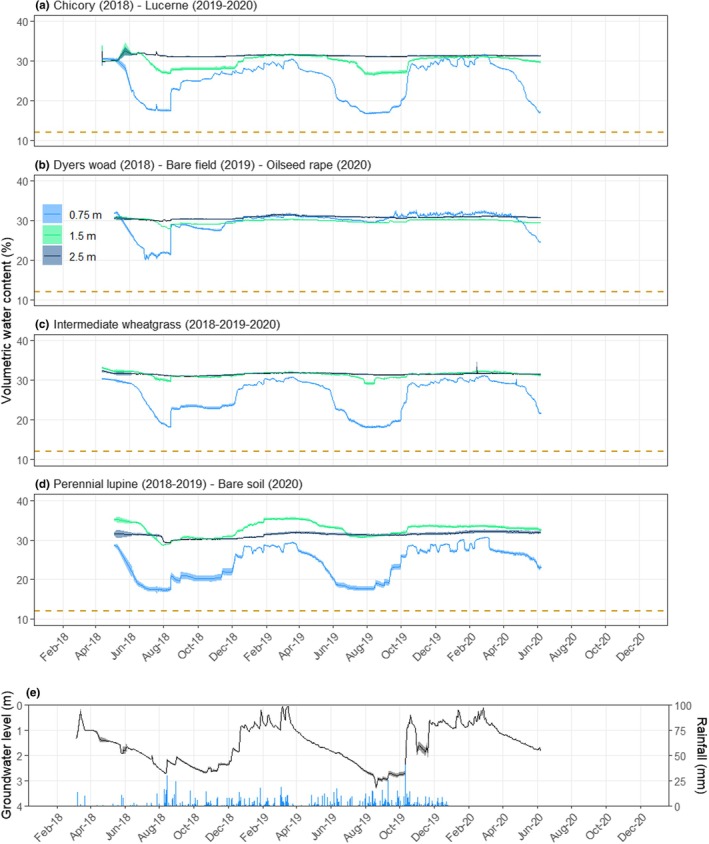
Soil volumetric water content evolution at 0.75 m (light blue), 1.5 m (green) and 2.5 m (dark blue) soil depth under four different crop rotations (a–d). Permanent wilting points for each depth were projected as dotted lines. Crop rotations are given in the figure's titles. Daily average value with SE (shaded area). Number of sensors per soil depth are as follows: *n*_a_ = 9, *n*_b_ = 3, *n*_c_ = 9 and *n*_d_ = 3. Daily groundwater level evolution (dark line) and rainfall pattern (bars) over the three seasons of cultivation (e). Groundwater level data were averaged over 4 wells and SE are presented with shaded area. Data cover three seasons of cultivation from April 2018 until October 2020. Lucerne and intermediate wheatgrass data 2018–2019 were already published in Clement *et al*. (2022).

TDR sensors gave us accurate measurements of the soil water content evolution over time. Soil water content followed a wetting and drying cycle, associated with seasonal differences in crop growth and rainfall patterns (Fig. 12). These differences also influenced the groundwater level which is characterized by relatively high ‘standing’ groundwater level in winter and low groundwater level (i.e. below 2.5 m depth) during the summer (Fig. 12e). Over the three growing seasons, all crops were able to deplete water from the 0.75‐m soil layer, with values reaching close to wilting point (i.e. 17% VWC; Fig. 12a–d). However, the rate of water depletion at this depth differs among crops. Perennial lupine, chicory and lucerne quickly depleted this soil layer, which almost all its available water used by early June. By contrast, slower rates of water depletion were observed for intermediate wheatgrass and dyers woad with soil water content reaching a value close to wilting point in July (Fig. 12b,c). Along with these results, perennial lupine, chicory and lucerne depleted substantially more water from the 1.5 m soil depth compared with intermediate wheatgrass and dyers woad. Indeed, at this soil depth, perennial lupine, chicory and lucerne induced *c*. a 5–6% drop in soil water content, whereas intermediate wheatgrass and dyers woad induced smaller changes of *c*. 2–3% VWC. However, no crops depleted this soil layer down to a value close to the wilting point. At 2.5 m soil depth, only perennial lupine induced a noticeable decrease of soil water content of 2% VWC, whereas no water use from this layer was shown for any of the other crops.

## Discussion

We went through three major steps to establish the facility: (1) installation of new equipment (minirhizotron and access tubes, water sensors, data loggers); (2) validating experimental procedures for root activity detection; and (3) streamlining the root quantification procedure using minirhizotron technique coupled with deep learning‐based segmentation.

### Challenges for installation in deeper soil layers

For both types of tubes, that is minirhizotron and access tubes, we faced challenges for insertion due to relatively high clay content (< 20%; Table S1) at the study site as well as high water table (*c*. 1 m) at the time of installation during the winter and early Spring in 2016–2017. The long tubes also strongly increase the friction while installing them into the holes after drilling. For minirhizotron tubes, therefore, it was unavoidable to make the holes larger than the tube diameter. This caused a gap between the upper soil surface and the tubes. We attempted to offset this issue by attaching raise‐blocks at the bottom side of each tube to ensure better soil‐tube contact at the upper side of the sloping tubes. The remaining gaps were filled with sand materials. However, this caused an artificial background on many images with the added sand, and for some tubes, the gaps were not filled (Figs 4, 5). This might have caused a low‐resistance path leading to an artificial increase in root growth (Rewald & Ephrath, 2013; and references therein). However, the minirhizotron tubes were inserted under the same protocol across the platform for a short period of time. Therefore, it is unlikely that the differences in deep root density between the crop species and observation timing were artefacts. For the metallic access tubes, we tackled this insertion issue by using sonic‐drilling – in which the resonating energy from a high‐frequency oscillation (e.g. 150 Hz) in the drill head ensures better penetration into the deep soil layers. After installation, we observed occasionally that some soil from upper opening areas fell into the tubes – which may have created an artefact for root growth by reducing root–soil contact.

### High‐throughput root measurement using root image analysis

One of the main advantages of the minirhizotron method is its capacity to measure gross root growth without interfering with the previously measured area/depth. We have further automated image capture by installing a programmable logic controller, which generates file names with useful meta‐data such as the plot number, location of camera (in terms of cable length projected) and timing of image capture as illustrated by Svane *et al*. (2019a). This enabled us to collect a large amount of data and process the images without much manual effort (e.g. re‐naming of the files).

Integrating the automated image capture with deep learning‐based analysis, we achieved a high efficiency of root data acquisition (*c*. 12 s per image). This certainly is a substantial improvement compared with manual root counting and annotation in terms of time saving. For example, *c*. 1 to 1.5 h of annotation time was required for 100 cm^2^ of root image area (Ingram & Leers, 2001), which now can be done in 1 min using our approach (even including the data capture and image processing time).

Our results showing the relationship between annotation time and root length correlation inform the RootPainter community on the trade‐offs of extended annotation for minirhizotron segmentation models. We found a large portion of model improvements to happen within the first 200 min, with extended annotation continuing to provide improvements but with diminishing returns (Fig. 3).

Some of our images captured the new root growth between the measurements taken at relatively short intervals. For example, lateral branching of lucerne roots at 0.35 m within 2 weeks was observed (Fig. 5a,b), which was successfully captured by the trained model showing the increase in root length from 11.21 cm to 15.57 cm per image. It was intriguing to see the rapid colour change of the main root from white to brown upon proliferation. Also, the imaging captured root growth of intermediate wheatgrass into depth, growing below 2 m within the growing season. This new occupancy of roots was observed between the measurements taken in July and September in 2018. Here, we show the images from June and September 2018 which better represent the location of imaging (Fig. 5c,d). This was also accurately determined by the trained model with the root length increasing from 0 cm to 7.3 cm. We were able to capture newly grown roots grown into the area at 1.21 m where dark roots of rosinweed were present from the growth in previous seasons (Fig. 5e,f). As a result, our approach was sufficiently accurate to capture the within‐season variation (May to August in 2017, 2018 and 2019) in root density of crop plants (Fig. 8), which makes consistent observation possible leading to a high‐time resolution dataset.

Although multispectral information was not used in this study, previous work has demonstrated the potential of spectral approaches for root classification. Multispectral imaging, including UV wavelengths has been used for root classification (Bodner *et al*., 2018; Svane *et al*., 2019a). The focus here was on automated root segmentation and data generation as a facility; however, future work shall exploit these capacities, with appropriate chemical and genetic validation, to classify root vitality and potentially enable species differentiation (Lombardi *et al*., 2025).

### Tracer‐based root activity measurement

Preinstallation of access tubes, insertion of ingrowth cores into the tubes containing tracer‐labelled soil made deep root nutrient uptake measurement possible up to 4.5 m soil depth. Two clear advantages of this CLT have been already addressed in detail by (Han *et al*., 2020). In short, this approach overcomes the disadvantage of the generic tracer injection, which can lead to movement of the injected aliquot depending on soil moisture content and rainfall (Hoekstra *et al*., 2014). Second, we were able to determine the roots of the plants that had access to the tracers. This ensures and validates that the root activity detected in the aboveground biomass was the result of roots growing into the tracer‐labelled soil of the ingrowth cores. This eradicates the potential discrepancy between root uptake and root density data, which can potentially occur under tracer injection methods (e.g. Da Silva *et al*., 2011). However, it is unclear how well the RLD within the ingrowth cores represent the RLD in the bulk soil around it. Our validation between aboveground tracer concentration and belowground root density showed a range of variation (*R* ^2^ values between 0.31 and 0.97) leading to an average of *R* ^2^ 0.67 (Fig. 11) confirming the reliability of the approach.

However, there was a discrepancy between the two root methods, namely, RLD by ingrowth core and pRLD by minirhizotron methods, despite the significant correlations (*P* ≤ 0.005). Several factors might have led to this disagreement between the two methods. First, the roots growing into the soil packed into the ingrowth cores may not represent those growing near the inserted minirhizotron tubes well. Several soil conditions differ (e.g. nutrient content; Table S1), and the roots in the bulk soil were typically well established before insertion of the ingrowth cores, while the roots in the ingrowth cores are the young roots which recently colonized the ingrowth‐core soil. Also, the 2‐month incubation time for ingrowth cores in this study might have been long enough for the freshly grown fine roots inside the ingrowth cores to undergo root turnover, whereas the roots visible to minirhizotron tubes might have been less susceptible to decomposition (Higgins *et al*., 2002), that is not completely exposed to soil microbes. In short, our results show that the two methods are not necessarily in agreement when determining root growth, and caution is required in interpreting the results when independently used.

### Sensor‐based deep‐water monitoring

The experimental setup in the DeepRootLab facility allows differentiation of crop water uptake in deep soil layers (i.e. > 1 m). Perennial lupine, chicory and lucerne were found to substantially use water down to 1.5 m soil depth and even deeper water uptake (i.e. down to 2.5 m) seems to occur under perennial lupine. Such results had also been for older lucerne crops, at the same location (Clément *et al*., 2022). The slower and more shallow water use by intermediate wheatgrass and dyers woad probably stemmed from different reasons. Dyers woad is a biennial crop, it produced deep roots, but matured early in the second season, terminating its water use in during June. Intermediate wheatgrass, on the other hand, is a perennial crop being more water spending. There are three drought escape strategies, (1) Escaping: usually achieved through shorter crop cycle (2) Water saving: through conservative use of water and (3) Water spending: using water and allocating more biomass to the growth of deep and abundant roots (Bodner *et al*., 2015; and references therein). Therefore, identifying crop water use strategies is of high importance to build cropping system that suits environmental conditions and maximize soil moisture capture (Blum, 2009). By allowing continuous measurements of root growth, water and nutrient uptake in deep soil layers, DeepRootLab constitutes a unique facility for the study of deep root growth and functions at the field crop and agricultural crop rotation level.

### Addressing research questions

#### Root growth dynamics

Among the tested crops, only a few (e.g. lucerne) have been reported for dynamics in root depth/penetration over multiple seasons. We observed an overall decrease in root penetration rate over time (Fig. 6). Nevertheless, looking at the yearly root growth dynamics (Fig. 7), it was clear that these perennial crops continue to develop deeper root systems over the years. However, the rates of root depth penetration were low compared with many annual crops (e.g. Thorup‐Kristensen, 2006). For many of the species studied, root depth penetration was also lower in the later years of development, as shown by the intercepts of the regression lines being in the range of 0.5–1.5 m for most crops, except for mugwort, rosinweed and comfrey, where intercepts were close to zero.

We also found a substantial variation in root penetration rate and total root depth between the species (Fig. 6). Our field site had a high water table (Fig. 12) – which might have restricted the root establishment of some of the crops, namely, lucerne, intermediate wheatgrass and perennial lupine. Indeed, a high water table has been shown to restrict root depth development of lucerne, which was limited to *c*. 2 m of maximum root depth under unfavourable subsoil conditions (Dolling *et al*., 2005). However, other perennials, such as rosinweed, comfrey and curly dock, reached a rooting depth of 3 m or more, indicating substantial species variation in root penetration capacity in waterlogged subsoil condition (Jacob *et al*., 2013).

In 2018 from March to August, a severe drought with high temperatures occurred at the study site (Fig. S1). During this period, the average temperature was 18.9°C (compared with 15.7°C for five previous seasons) and the precipitation received was 48 mm, which was merely 23% of the 5‐yr average (209 mm; Sears *et al*., 2021). Accordingly, we observed a marginal increase in deep rooting and root density for perennial lupine and mugwort and even a decrease for lucerne and curly dock from the last measurements in 2017 (June–October) to the first measurement in 2018 (May–June). This observation contradicts the general notion that plants tend to invest more in root growth as a response to water limitation.

However, unlike the crops in need of developing deeper roots to reach the subsoil water, our perennial crops, except comfrey, already had access to the underground water. During the time of drought in 2018, the reported groundwater levels were *c*. 1–2 m from April to July (Clément *et al*., 2022; Fig. 12E). Such conditions can lead to the paradoxical co‐occurrence of drought stress in the topsoil and waterlogged conditions at depth, which is common in lowland or shallow groundwater systems. Therefore, the reduction in root density from 2017 to 2018 may reflect hampered root associated with this combined growth (Sheaffer *et al*., 1988; Kuster *et al*., 2013; Herzog *et al*., 2016).

#### Deep root‐mediated nutrient uptake – crop comparison

We conducted three experiments comparing root growth and nutrient uptake potential (tracer labelling) of crop species. According to the minirhizotron imaging and analysis, perennial crops exhibited deeper rooting and higher root density than annual crops (e.g. chicory vs winter rye in Exp 1; curly dock vs winter wheat in Exp 3). Taproot‐dominated crops (e.g. perennial lupine) grew more roots at depth, whereas fibrous root‐dominated crops (e.g. intermediate wheatgrass, tall fescue and winter wheat) established more roots in shallower layers. The tendency for deeper root growth by crops with high root diameter is attributed to their capacity to overcome mechanical resistance in the subsoil layers upon penetration (Materechera *et al*., 1992). This interpretation is consistent with recent studies showing that root penetration in mechanically strong soils is driven by anatomical reinforcement. Schneider *et al*. (2021) demonstrated that multiseriate cortical sclerenchyma (MCS) increases root tensile strength and penetration under mechanical impedance, while Lopez‐Valdivia (2025) showed that mechanically reinforced roots more effectively penetrate compacted soils and access deeper soil domains.

When it comes to root activity, annual crops and relatively younger crops (chicory, dyers woad and curly dock) showed stronger capacity to grow fresh roots into ingrowth cores leading to a greater ^15^N uptake. Older crops with higher root density at depth observed via minirhizotron tubes tended to grow fewer roots into the ingrowth cores and exhibited weaker ^15^N enrichment. Rosinweed, as an older crop, showed greater N uptake capacity at 2.2–2.8 m.

Choice of management (e.g. early sowing and subsoil structurization) and genotypes (e.g. with high root penetration rate) can often lead to deeper and denser root systems accompanied by improved nutrient uptake potential (Han *et al*., 2015; Rasmussen & Thorup‐Kristensen, 2016). It can also be driven by the differences in crop demands at the time of observation. Our data cannot address how the actual interactions between uptake capacity and demand contributed to the observed results. Nevertheless, a few points can be addressed. First, annuals (winter rye and winter wheat) during the spring to summer (March–July), exhibited greater N uptake at the topsoil layer and shallower subsoil layers than did the old perennials (lucerne and rosinweed) despite their smaller root density. This is an intense growing period for newly established crops, which must assimilate N within a very short period. On the contrary, perennials can remobilize N from the previous year and have a longer season for N uptake. However, the deep‐rooted perennials (e.g. rosinweed and curly dock) showed greater N uptake capacity at deeper soil layers when compared with annuals (2.2–2.8 m). This demonstrates that deep‐rooted perennials, have better access to deep‐placed nutrients, improving overall nutrient use efficiency and hold potential for preventing nitrate leaching loss (Thorup‐Kristensen, 2006; Van Tassel *et al*., 2017).

### Conclusions

We have successfully established a field facility in which a variety of measurements on deep root growth and function can be made. It allows the relatively easy and repeated study of root growth and function to great depth under field conditions, by using preinstalled minirhizotrons, access tubes and soil water sensors. The extent and design of the facility allow experiments with up to 48 plots, allowing several species or treatments to be studied in well‐replicated studies. The facility is an ideal platform for conducting studies at deep soil layers in the field with a capacity for generating statistically and biologically meaningful results.

## Competing interests

None declared.

## Author contributions

EH prepared the manuscript and CC, WC, AGS, DBD and KTK contributed to the writing. EH trained the model for automatic root segmentation and conducted all the ingrowth‐core experiments. CC installed the TDR system and conducted the experiment on crop water use. AGS created the RootPainter software and provided technical advice on training models for root detection. KTK and DBD conceptualized the facility establishment. EH, KTK and DBD contributed to the experimental designs.

## Disclaimer

The New Phytologist Foundation remains neutral with regard to jurisdictional claims in maps and in any institutional affiliations.

## Supporting information


**Fig. S1** Weather conditions at the study site in Taastrup, Denmark.
**Table S1** Physical and chemical soil characteristics at the study site and for the ingrowth cores.
**Table S2** Dates of minirhizotron imaging.
Please note: Wiley is not responsible for the content or functionality of any Supporting Information supplied by the authors. Any queries (other than missing material) should be directed to the *New Phytologist* Central Office.

## Data Availability

The root image data and trained model can be downloaded from Zenodo doi: 10.5281/zenodo.15213661.
