# Supplementary material for: Deep roots through time and crops: insight from five seasons at DeepRootLab
Source: New Phytol. 2026 Mar 13;250(4):2670–88. doi: 10.1111/nph.71065 (PMC13103413; doi:10.1111/nph.71065)
Supplement: Supplementary file 1 — Fig. S1 Weather conditions at the study site in Taastrup, Denmark. Table S1 Physical and chemical soil characteristics at the study site and for the ingrowth cores. Table S2 Dates of minirhizotron imaging. Please note: Wiley is not responsible for the content or functionality of any Supporting Information supplied by the authors. Any queries (other than missing material) should be directed to the New Phytologist Central Office. [file NPH-250-2670-s001.pdf]

New Phytologist Supporting Information

Article title: Deep roots through time and crops: Insight from five seasons at DeepRootLab

Authors: Eusun Han, Corentin Clément, Weronika Czaban, Abraham George Smith, Dorte Bodin Dresbøll, Kristian Thorup-Kristensen

Article acceptance date: 11 February 2026

Supporting Information

Table S1. Physical and chemical soil characteristics at the study site and for the ingrowth-cores 2

Table S2. Dates of minirhizotron imaging 3

Figure S1. Weather conditions at the study site in Taastrup, Denmark 4

Table S1. Physical and chemical soil characteristics at the study site and for the ingrowth-cores

Soil type	Soil depth	pH	Clay	Silt	Fine sand	Coarse sand	Bulk density	P	K
	(m)		(%)				(g cm^{-3})	(%)	
Ingrowth-core soil		8.1	12.5	12.4	45.5	28.9		0.004	0.041
Field soil	0-0.25	7.6	13.0	15.2	42.3	27.8	1.56	0.042	0.110
	0.25-0.75	7.8	20.2	12.9	40.3	26.1	1.64	0.013	0.084
	0.75-1.5	4.5	19.9	16.3	37.8	25.9	1.76	0.006	0.065
	1.5-3.0	8.2	19.3	18.9	36.6	25.1	1.77	<0.004	0.074
	3.0-4.5	8.1	19.0	25.9	33.1	21.7	1.77	0.004	0.111

Table S2.Dates of minirhizotron imaging

	n	2016	2017	2018	2019	2020	Campaigns
Lucerne	6	Aug 9[Y]	May 4[S]<br>Jun 6[S,E], 20[E]<br>Jul 5[S,E]<br>Aug 8[Y,S]	May 31[S]<br>Jun 5[S]<br>Jul 5[Y,S]<br>Sep 9[S]	May 7[S], 30[S]<br>Jul 4[S]<br>Aug 14[Y,S]		16
Intermediate wheatgrass	9	Aug 24[Y]	Feb 23[S]<br>Apr 3[S]<br>Jun 16[Y,S]	May 31[S]<br>Jun 5[S,E]<br>Jul 5[Y,S,E], 20[E]<br>Sep 9[S]	May 7[S], 30[S]<br>Jul[S],<br>Aug 19[Y,S]		13
Perennial lupine	3	Aug 24[Y]	Apr 4[S]<br>Jun 16[Y,S]<br>Aug 9[S]<br>Sep 5[S]	Jun 5[S,E], 20[Y,S]<br>Jul 10[S,E], 27[E]	May 8[Y]		9
Mugworth	3	Sep 6[Y]	Apr 5[S]<br>Jun 16[S]<br>Jul 19[Y]<br>Aug 9[S]	Jun 6[S], 20[S]<br>Jul 10[Y,S], 27[S]	May 10[Y]		10
Rosinweed	6	Sep 1[Y]	Mar 6[S]<br>Apr 5[S]<br>May 24[S]<br>Jun 19[Y,S]	Jun 6[Y]	May 7[S,E], 20[E]<br>Jun 4[S,E]<br>Jul 4[S]<br>Aug 13[Y,S]		11
Comfrey	3		Apr 4[S]<br>Jun 16[Y,S]<br>Aug 9[S]	Jun 6[Y,S], 20[S]<br>Jul 9[S], 27[S]	May 10[Y]		8
Curly dock	6		Jul 18[Y,S]<br>Aug 11[S]<br>Sep 11[S]<br>Oct 10[S]	Jun 6[Y]	May 7[S,E], 20[E]<br>Jun 4[S,E]<br>Jul 4[S]<br>Aug 13[S]		10
Tall fescue	6				Jul 4[Y,S]<br>Aug 13[S]	Apr 28[Y]	3
Chicory	9		Jun 6[E], 20[E]<br>Jul 5[E]				3
Dyers woad	9			Jun 5[E]<br>Jul 5[E], 20[E]			3
Winter rye	6		Jun 6[E], 20[E]<br>Jul 5[E]				3
Winter wheat	6				May 7[E], 20[E]<br>Jun 4[E]		3

Y: Yearly dynamics
S: Seasonal dynamics
E: Experiment

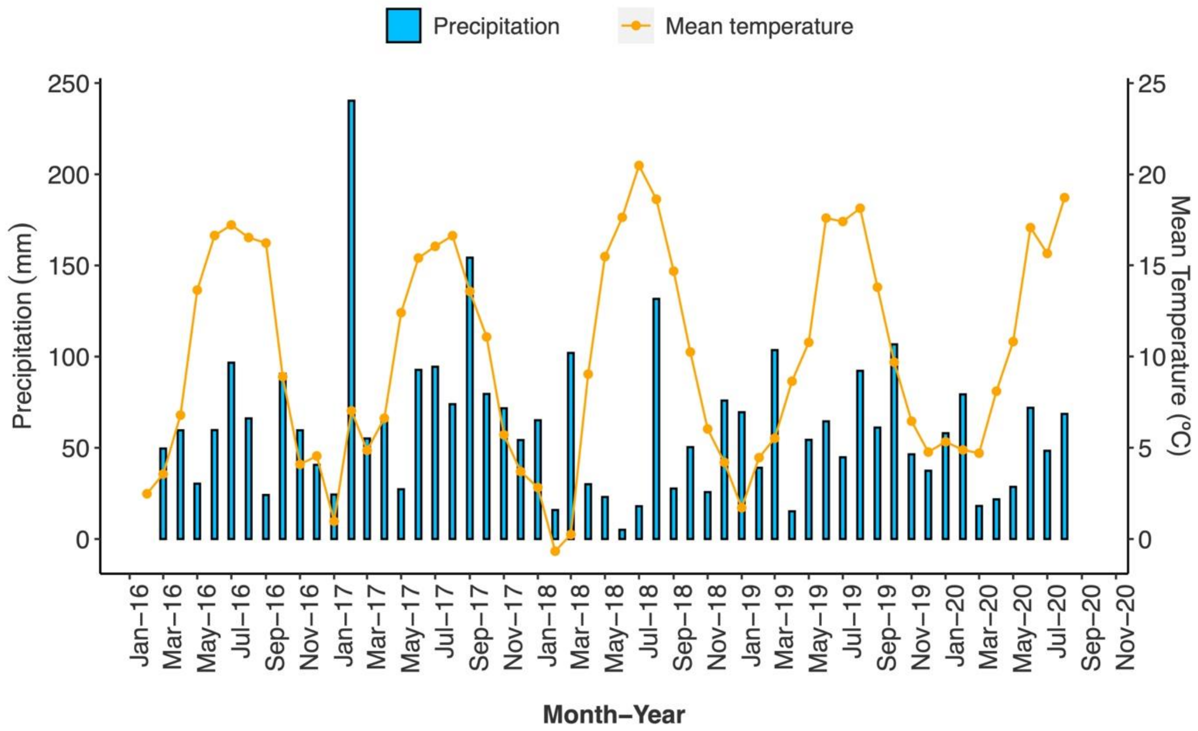

Figure S1. Weather conditions at the study site in Taastrup, Denmark
